# Let My People Go (Home) to Spain: A Genealogical Model of Jewish Identities since 1492

**DOI:** 10.1371/journal.pone.0085673

**Published:** 2014-01-22

**Authors:** Joshua S. Weitz

**Affiliations:** School of Biology and School of Physics, Georgia Institute of Technology, Atlanta, Georgia, United States of America; Universitat Pompeu Fabra, Spain

## Abstract

The Spanish government recently announced an official fast-track path to citizenship for any individual who is Jewish and whose ancestors were expelled from Spain during the inquisition-related dislocation of Spanish Jews in 1492. It would seem that this policy targets a small subset of the global Jewish population, that is, restricted to individuals who retain cultural practices associated with ancestral origins in Spain. However, the central contribution of this manuscript is to demonstrate how and why the policy is far more likely to apply to a very large fraction (i.e., the vast majority) of Jews. This claim is supported using a series of genealogical models that include transmissible “identities” and preferential intra-group mating. Model analysis reveals that even when intra-group mating is strong and even if only a small subset of a present-day population retains cultural practices typically associated with that of an ancestral group, it is highly likely that nearly all members of that population have direct genealogical links to that ancestral group, given sufficient number of generations have elapsed. The basis for this conclusion is that not having a link to an ancestral group must be a property of all of an individual’s ancestors, the probability of which declines (nearly) superexponentially with each successive generation. These findings highlight unexpected incongruities induced by genealogical dynamics between present-day and ancestral identities.

## Introduction

Present-day Jews predominantly self-identify as either Sephardic or Ashkenazi. Origins of Sephardic Jews are generally attributed to the Jewish community based in Spain and Portugal that was expelled from the Iberian peninsula in the late 15th century, whereas Ashkenazi Jews generally attribute their origins to Central and Eastern Europe, pre-dating the expulsion [1], [2]. These divisions are, at least culturally, considered to be long-standing, for example, the protagonist of the classic 19th century farce “The King of Schnorrers” (which is set in the late 18th century) – Manasseh Bueno Barzillai Azevedo da Costa – reacts in horror at the prospect of his daughter marrying an Ashkenazi, rather than a Sephardic, Jew: “A Sephardi cannot marry a Tedesco [Ashkenazi]! It would be a degradation” [3]. The conditions for the fast-track naturalization announced by the Spanish government in November 2012, follow along these traditional designations of ethnic identity: (i) the petitioner must be Jewish; (ii) the petitioner must “certify” their Spanish Jewish origins. Indeed, the announcement and subsequent media coverage of this change to Spanish civil law highlighted its intended target to be self-identified Sephardic Jews [4], [5] – a minority of the global Jewish population. It would seem that being a direct descendant of an individual expelled from Spain in 1492 would be a necessary condition for fast-track naturalization, however, it remains unclear as to whether this will be a sufficient condition. Here, the following question is asked: to what extent should *any* Jew living today expect to have one (or more) Jewish ancestors expelled from Spain in 1492.

Although identities may indeed be exclusive and even strongly retained inter-generationally, this does not preclude the fact that individuals of one identity may have one (or more) ancestors of a different identity. Irrespective of identity, the number of ancestors that any individual has grows quite rapidly, exponentially at first and then increasing (albeit at a slower-than-exponential rate) with each successive prior generation. Hence, a present-day individual that self-identifies as Jewish would have a direct genealogical link to the expelled Spanish Jewish community if one (or more) of their deceased forebears in 1492 was a member of that community. Here, the main contribution is to develop a simple genealogical model (and intuition) to explain how having ancestors of diverse “types” is extremely common even when cross-“type” mating is rare.

## Results

### Model of Genealogical Dynamics with Assortative Mating

Consider the genealogical dynamics of a population of 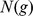 individuals where 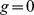 denotes the ancestral population of interest (e.g., the number of Jews living in Spain in 1492) and 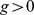 denotes each successive prior generation such that 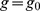 denotes the present. In this model, given an initial population size, 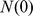, then 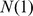 pairs of parents are selected at random from the 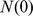 individuals present in the 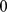-th generation, forming a history of genealogical links between each generation. The value of 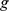 is then incremented iteratively from 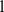 to 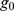. In this model, individuals can be selected more than once and no information on male/female identities are retained. These dynamics correspond to the forward version of standard models of genealogical dynamics with sexual reproduction [6], [7]. Statistical properties of genealogical dynamics at the population scale have previously been found to be highly robust to this apparent lack of realism [8]–[10].

Next, consider a modified version of the standard model of genealogical dynamics with sexual reproduction to distinguish between individuals with two exclusive traits, type 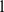 or type 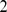, e.g., Sephardic (type-1) or Ashkenazi (type-2) These traits need not be genetically encoded. In this framework, each generation can be described in terms of a population-wide distribution of traits, 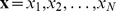 where 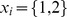. The fraction of type-1 individuals is denoted as 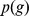. Individuals of different traits may prefer to mate with individuals with the same trait. A generalized mating preference parameter 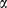 can account for preferential mating, such that the 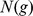 parents of individuals in generation 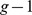 will be selected from a multinomial distribution with probabilities: 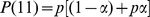, 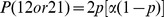, and 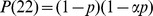, where 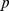 is the proportion of type-1 individuals in generation 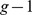. Hence, when 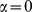, mating occurs exclusively amongst individuals of the same type, i.e., 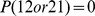. When 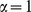, mating probabilities depend on population proportions exclusively, i.e., 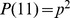, 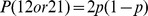, and 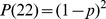. Intermediate values of 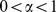 provide a continuum between these limits, consistent with models of varying degrees of assortative mating in population genetics. The model further presumes that children of two type-1 parents self-identify as type-1 individuals, and that children of two type-2 parents self-identify as type-2 individuals. Finally, the model assumes that children of a type-1 and a type-2 parent self-identify as either type-1 or type-2 with equal probability.

### Individuals have Ancestors with Multiple Identities, even when Mating is Strongly-preferred among Individuals of the Same Identity

**Figure 1:**
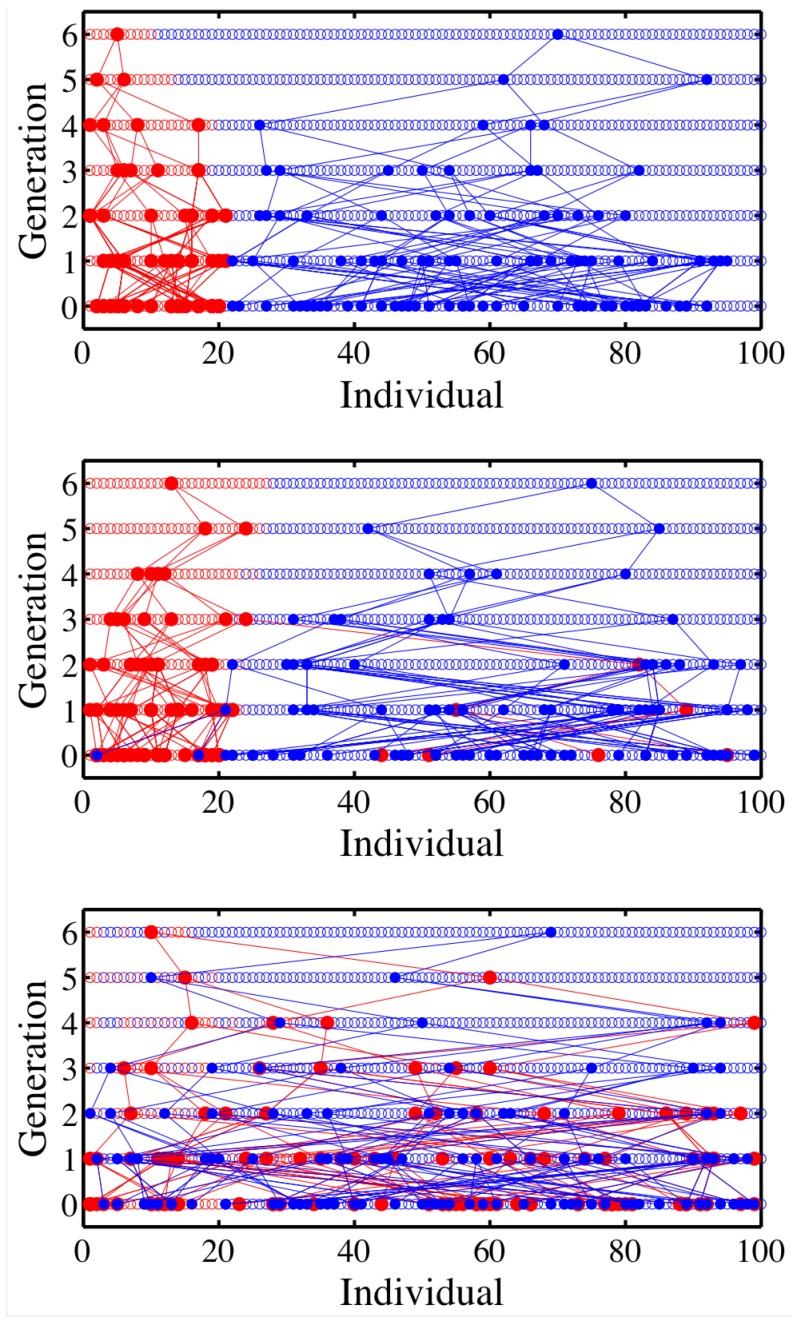
Genealogical history with mating preferences. Each panel illustrates the ancestors of two focal individuals given 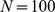 and 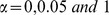 (top-middle-bottom). Initially, at 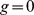, there are 20% type-1 individuals (red circles) and 80% type-2 individuals (blue circles). Ancestors are denoted by red and blue lines, respectively. When viewing the process retrospectively from the present-day (top line in each panel, 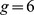), it is apparent that all ancestors share the same identity as the focal two individuals when 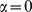. However, when 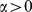 then one (or more) ancestors may have a different identity than that of the focal individual.

Trait-associated genealogical dynamics were simulated in the modified model, described above, for three values of 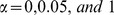 (see Figure 1). When 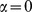, then the identities of ancestors must match that of present-day individuals, since there is no reproduction among individuals of different identities. In this limit, the fraction of present-day individuals with no type-1 ancestors remains relatively constant (near 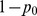). Whereas, when 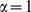, then reproduction occurs irrespective of identity. Hence, it is highly likely that individuals have ancestors of both identities. Notably, the same phenomenon is observed even when preferences are strong for intra-type reproduction, e.g., when 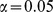. The reason is occasional (even rare) instances of cross-identity reproduction among an exponentially growing number of ancestors (and matings) lead to frequent instances of type-1 individuals with type-2 ancestors and vice-versa. Moreover, once a type-1 individual has at least one type-2 ancestor, then the mating preferences reinforce this history, ensuring that type-1 individuals often have many type-2 ancestors (and vice versa). These exploratory simulations reveal that individuals can have ancestors with identities other than their own, even when individuals strongly prefer to reproduce with individuals of the same identity.

### The Likelihood of having an Ancestor with an Identity Different than One's Own Increases (Nearly) Super-exponentially with Prior Generations

**Figure 2:**
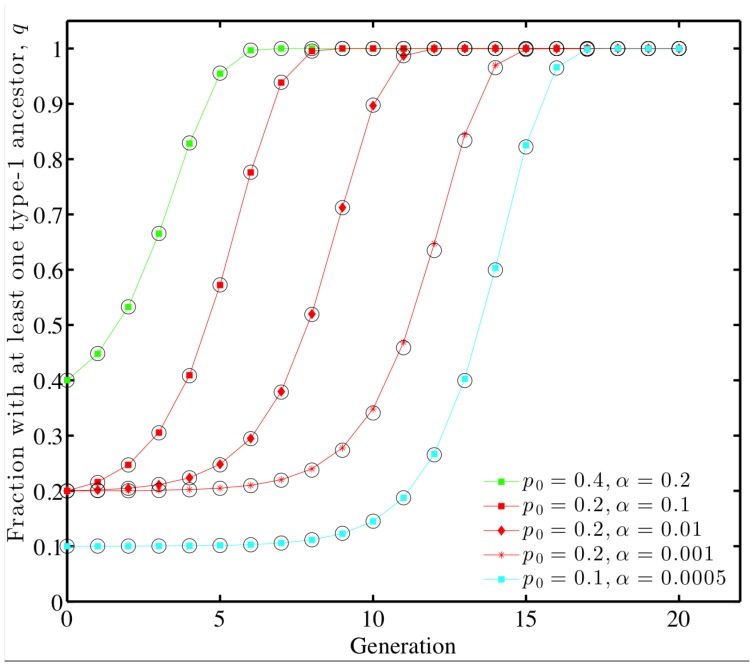
Rapid increase in frequency of genealogical link to a previously minority identity. Solid lines with symbols denote theory of Eq. (1) (colored based on initial frequency of type-1 individuals) while circles denote simulation. The simulated population has 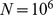 individuals.

Introducing the concept of *genealogical identity* will prove useful to explore the phenomenon of having ancestors with identities different than that of a focal individual, Let 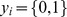 represent whether or not any of a focal 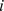-th individual's ancestors had a particular identity of interest. In this study system, 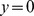 denotes that none of an individual's ancestors was of type-1, whereas 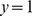 denotes that at least one ancestor of a focal individual was of type-1. Hence, the state of an individual can be described in terms of 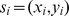. Due to the definition of identity transfer in the model, there are three possible states of individuals: (i) 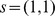 - a type-1 individual with at least one type-1 ancestor; (ii) 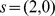 - a type-2 individual with no type-1 ancestors; (iii) 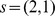 - a type-2 individual with at least one type-1 ancestor. The genealogical identity 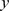 of children is equal to 1 when either parent is of type 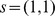 or 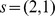 and equal to 0 only when both parents are of type 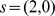. Analysis of genealogical dynamics yield a closed form prediction for the fraction of individuals with at least one type-1 ancestor:
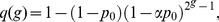
(1)(see derivation in the Methods). The fraction of individuals with at least one type-1 ancestor rapidly approaches one, given that the probability of having no type-1 ancestors declines (nearly) super-exponentially. This prediction can be evaluated for different values of 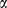 and 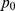 in a large population simulation where 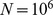. Quantitative agreement is found even when 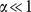 (see Figure 2). Indeed, even when the vast majority of sexual reproductions occur between individuals of the same identity as occurs when 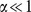, the fraction of individuals with at least one type-1 ancestor, 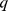, converges rapidly from 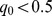 to 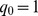.

### Nearly All Present-day Jews are Likely to have at least One (if not many more) Ancestors Expelled from Spain in 1492

**Figure 3:**
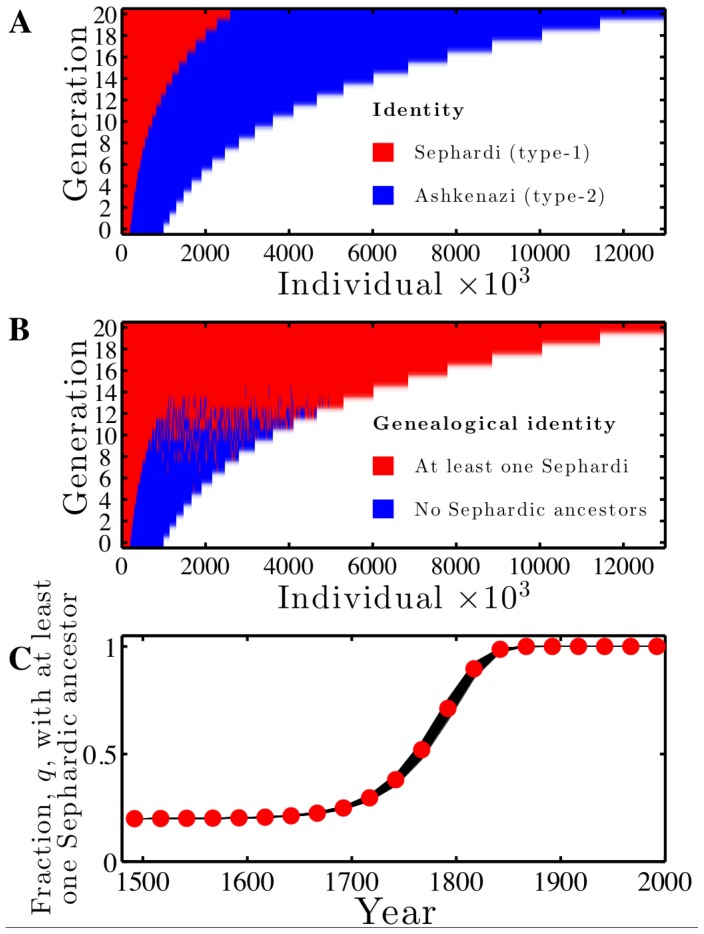
Nearly all modern-day Jews are likely to have many ancestors expelled from Spain in 1492. Genealogical dynamics are simulated using 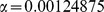 in a population of initial size 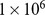 at generation 0 that is of size 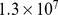 at generation 20. The choice of 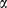 corresponds to a relative preference of in-group mating of 1000∶1 relative to out-group mating. (A) Population-state of identities, 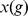, where the identities (red for Type-1, 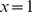, and blue for Type-2, 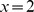) are displayed for each individual (column) in each generation (row). (B) Population-state of genealogical identities, 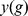, where the identities (red for 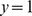 and blue for 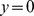) are displayed for each individual (column) in each generation (row). (C) Comparison of theoretical prediction (red circles) of the fraction of individuals with at least one Sephardic ancestor, 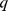, with 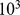 simulations (all variation contained in black shaded region). The identity information in panels (A) and (B) are downsized by a factor of 1000∶1 for the purposes of visualization.

The modified genealogical model with mating preferences can be evaluated in a parameter regime inspired by that of transmission of Sephardic and Ashkenazi identities. This regime includes the assumptions that 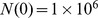 and that 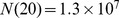, spanning an approximately 500 year time frame, corresponding to approximately 20 generations [11], [12]. Further, the application of the model assumes that the Sephardic community was approximately 20% of the global Jewish population in 1492 [11]. Mating preference data spanning this historical period is not available in a comprehensive fashion. Instead, consider a highly conservative (and highly biased) mating scenario where 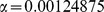, corresponding to a 1000∶1 relative likelihood given one type-1 individual to mate with another type-1 individual rather than a type-2 individual. Note that analysis of the 1995 Israeli national census finds that marriages between Sephardic and Ashkenazi individuals is reported to occur approximately 10-fold less frequently than within-type marriages [13]. Such modern estimates suggest that strong inter-type preferences continue to have persisted into the 20th century. Simulations reveal that, despite an extreme preference for in-group mating, it takes only 15 generations for all individuals to have at least one direct genealogical link to a Sephardic in the 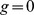 generation (see Figure 3B). This occurs despite the fact that Sephardic identity remains a minority throughout the simulation (see Figure 3A). The frequency of individuals with at least one type-1 ancestor, 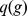, agrees with theoretical predictions (see Figure 3C), further substantiating the generality of the present mechanism for the spread of genealogical identity in constant and in varying populations. These results are highly robust to changes in initial population fractions, 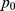, population sizes 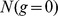 and 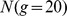, and mating preferences 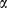.

**Figure 4:**
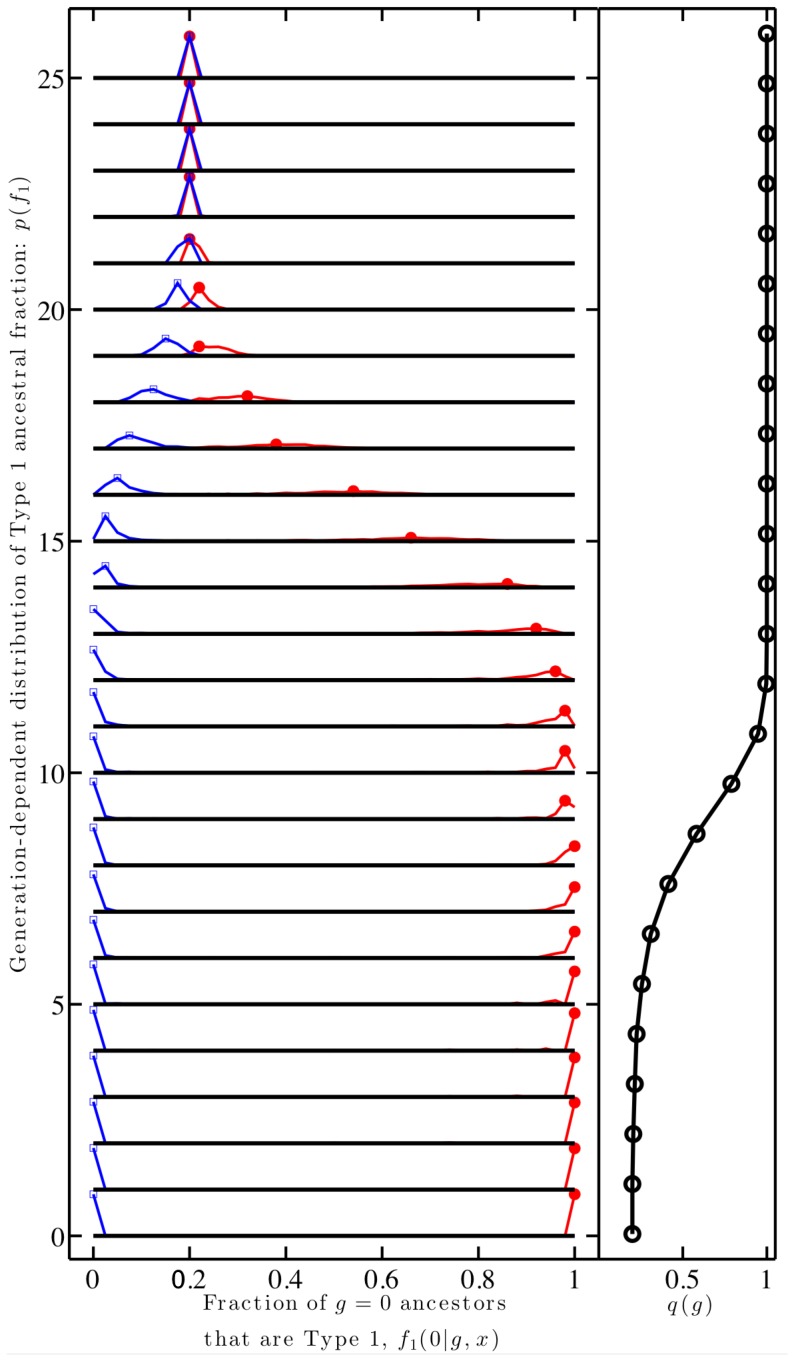
Individuals have many ancestors with different identities than their own. The results are from a simulation of a population with 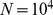, 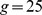, 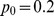 and 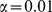. (Left) Fraction of ancestors in 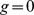 that are type 1, for individuals who at generation 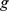 self-identify as type 1 (red) or type 2 (blue); (Right) Fraction of individuals with at least one type-1 ancestor, 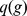. Generations increase from 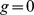 to 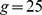 along the y-axis in both panels.

Claims regarding the diversity of ancestral identities can be extended using the same model framework. Specifically, the model predicts that individuals are not only likely to have at least one ancestor of an identity different that their own (as shown in Figures 2 and 3), but are likely to have *many* such ancestors. To illustrate this concept, consider a type-1 individual in generation 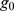 who has one type-2 ancestor in generation 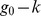. Then, the type-1 individual would be a direct genealogically descendant of many type-2 ancestors in generation 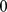 via this (rare) link, subsequently reinforced by assortative mating. A quantitative metric can be introduced to characterize this phenomenon: 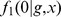 - the fraction of ancestors in generation 0 who are of type 1 for an individual living in generation 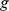 whose identity is 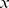. Simulations reveal that 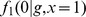 converges to that of 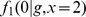 over time. This implies that individuals of different identities both share many, and eventually, all ancestors of a focal identity (see Figure 4). This claim is consistent with prior analysis of panmictic populations in which there is a rapid (i.e., scaling independently of population size) and recent (i.e., scaling with the logarithm of population size) transition, in terms of generations, over which two randomly chosen individuals are likely to switch from sharing very few to nearly all of their ancestors [7].

## Discussion

In summary, a series of simplified genealogical models have been proposed and analyzed in which individuals retain a set of identities. Nearly all model variants lead to dynamics in which present-day individuals who identify with one identity have at least one, and typically many, ancestors of a different identity in a relatively recent generation. As is apparent, the model formulation is generic rather than specifically parameterized for the detailed and complex structure of present-day and historical Jewish populations - which inspired the present analysis. Hence, extensions are warranted that include population structure, mating with other “groups” (e.g., those who do not transmit or retain the cultural practices associated with the identity), loss of identity, and other demographic structure that may change both the baseline expectation and variation for identity dynamics as described by the present model. Nonetheless, given the super-exponential nature of the process, it would require significant changes to the model structure to substantively modify the overall conclusion: given occasional mating between individuals of different identities, then a small number of generations is likely required for individuals to have a subset of their ancestors with identities different than their own. This notion is strongly consistent with landmark work on the genealogical ancestry of all living humans [9] and population genomics research on the related ancestry of individuals from seemingly disparate European populations [14].

Returning to the inspiration for this model, note that the Spanish government's fast track to citizenship was ostensibly meant to target Sephardic Jews, a minority of the global Jewish population. Extrapolating from the model analysis, it would seem that, to the contrary, the far more likely baseline hypothesis is that the vast majority of present-day Jews have one or more direct genealogical forebears in the Jewish community expelled from Spain in 1492. The current analysis does not consider the policy implications of such a hypothesis. However, it is worth noting that the fast-track naturalization process announced in November 2012 has yet to be implemented (as of May 2013) [15] and that Portugal has also recently announced a similar policy modeled on the Spanish framework [16]. Instead, the model can be used to point out that policies linked to the identity of (distant) ancestors should be approached cautiously, given that the number of ancestors grows (nearly) exponentially. As a consequence, the identity-associated characteristic of ancestors need not be congruent with the identities of present-day individuals. In the words of Manasseh Bueno Barzillai Azevedo da Costa, the protagonist of The King of Schnorrers [16], “Never before have I sat at the table of a Tedesco [Ashkenazi] – but you – you are a man after my own heart. Your soul is a son of Spain.”

## Methods

The dynamics of genealogical identity can be derived as follows. Consider a population of individuals each with state 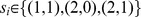. Denote 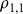, 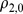 and 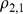 as the population wide probability of a randomly selected individual to have the state denoted in the subscript, e.g., 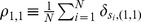. For convenience, define the time-varying fraction of type-2 individuals with no type-1 ancestors as 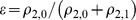. Initially, 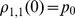, 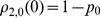, and 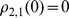. In generation 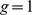, 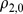 is equal to the fraction of individuals both of whom had type-2 parents, i.e., 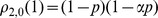, and 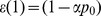. In subsequent generations, some type-2 individuals themselves have type-1 ancestors. Of the type-2 individuals born to parents who are both type-2, only a fraction, 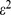, are a product of reproduction involving parents with no type-1 ancestors. Hence, 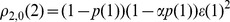. Recall that the genealogical dynamics modeled here preserves the fraction of typed individuals, on average, a result leveraged by assuming that 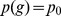. Therefore, the predicted discrete-time population dynamics of the system can be written, for 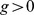 as:
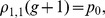


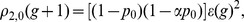


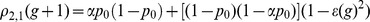



In this framework,
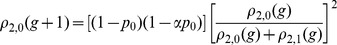



But, recalling that 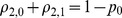, this can be simplified as:
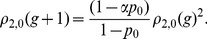
(2)


Eq. 2 can be solved recursively, implying that the subpopulation of type-2 individuals with no type-1 ancestors declines superexponentially so long as 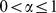: 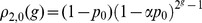. Finally, the fraction of individuals with at least one type-1 ancestor is 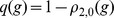, as stated in the main text. Simulation code used to generate figures in this manuscript is available as File S1.

## Supporting Information

File S1 **Software for simulating assortative ancestries.**
(ZIP)
